# Optimization of Castor Oil-Based Ion Selective Electrode (ISE) with Active Agent 1,10-Phenanthroline for Aqueous Pb^2+^ Analysis

**DOI:** 10.3390/membranes12100987

**Published:** 2022-10-11

**Authors:** Khairun Nisah, Rahmi Rahmi, Muliadi Ramli, Rinaldi Idroes, Sagir Alva, Muhammad Iqhrammullah, Eka Safitri

**Affiliations:** 1 Graduate School of Mathematics and Applied Sciences, Universitas Syiah Kuala, Banda Aceh 23111, Indonesia; 2 Department of Chemistry, Faculty of Sciences and Technology, Universitas Islam Negeri Ar-Raniry, Banda Aceh 23111, Indonesia; 3 Department of Chemistry, Faculty of Mathematics and Natural Sciences, Universitas Syiah Kuala, Banda Aceh 23111, Indonesia; 4 Department of Pharmacy, Faculty of Mathematics and Natural Sciences, Universitas Syiah Kuala, Banda Aceh 23111, Indonesia; 5 Mechanical Engineering Department, Faculty of Engineering, Universitas Mercu Buana, West Jakarta 11650, Indonesia; 6 Department of Life Science and Chemistry, Jacobs University Bremen, 28759 Bremen, Germany

**Keywords:** potentiometry, ISE, polyurethane, 1,10-phenanthroline, lead

## Abstract

This research has successfully fabricated ion selective electrode (ISE) for Pb^2+^ using castor oil (*Ricinus communis* L.)-based polyurethane (PU) membrane with 1,10-phenanthroline as the active agent. The sensitivity of the Pb^2+^ ISE obtained is 27.25 mV/decade with a linear range of [Pb(NO_3_)_2_] of 10^−10^–10^−5^ M and a coefficient of determination (R^2^) of 0.959. The system response reaches stability after 25 s of measurement. The Pb^2+^ has a detection limit of 10^−10^ M and gives a stable response at pH 7–8 with a 15-day lifetime. The investigation of the selectivity of the ISE was performed using the mixed solution method with log Kij values of <1. The selectivity order of Pb^2+^ ISE against the foreign ions is Ag^2+^ > Ca^2+^ > K^+^ > Mg^2+^ > Cu^2+^ > Fe^3+^ > Cr^3+^> Zn^2+^ > Cd^2+^. The Pb^2+^ ISE shows acceptable reproducibility and repeatability with standard deviation values of 0.065 and 0.0079, respectively. Fourier transform infrared (FT-IR) spectra confirmed that 1,10-phenanthroline was responsible for the formation of the Pb^2+^ ion entrapment via complexation. Other characterizations (crystallinity, micro-surface morphology, and mechanical strength) suggest the degradation of the membrane structure integrity after the application. The analysis results of Pb levels using the Pb^2+^ ISE in artificial and wastewater samples were not significantly different from the atomic absorption spectroscopy (AAS) measurement.

## 1. Introduction

The consequence of improved living standards is followed by industrial growth, where increased pollution, particularly heavy metals, is expected. Lead (Pb) exposure could be harmful to the ecosystem and, more importantly, humans. This is owing to the fact that Pb could be bioaccumulated in bone tissue and damage neural functions deriving from its neurotoxicity [1]. Children have been found to be a higher risk group for Pb intoxication [2]. Toxicity of Pb is a serious problem because this metal is extensively used in industry, such as pipe [3] and paint manufacturers [4]. In addition, Pb is also used to increase the octane number of premium fuel [5]. Therefore, Pb should be analyzed on a routine basis to monitor the pollution level in order to prevent its deleterious effects. Based on the stated problem, a quick, cheap, sensitive, and accurate analysis of Pb is required. 

Pb could be analyzed using conventional and instrumental methods. The instrumental method include ion selective electrode (ISE), which has been recognized for its high sensitivity because it could measure Pb content as low as 10^−10^ M concentration units [6]. Determination of Pb could also be carried out by spectrophotometry [7], atomic absorption spectrometry (AAS) [8], and X-ray fluorescence [9]. In general, methods requiring equipment such as UV-Vis spectrophotometer and AAS are complicated and could only be operated by skilled personnel. Besides, the analysis requires a long time, especially for the sample pre-treatment.

Potentiometry could be an alternative for Pb analysis, in which this method could measure low concentrations, have properties of being cheap and accurate, and does not require sample pre-treatment [10]. Parts of the equipment used for the Pb detection is ion selective electrode (ISE Pb^2+^). The ISE consists of a matrix and active components (ionophore) that is responsible for ISE performance, such as the selectivity of the desired analyte [11]. This tool has been developed in the last few decades, but its development is slower compared to other electrometry methods such as voltammetry. This could be due to the limitation on the available active agents and compatible membrane in order to yield satisfying results. Various matrix and active agents have been employed to construct Pb^2+^ ISE, such as glass multi-component chalcogenide (ChG), and GeSe2-PbSe-PbTe, respectively [12]. Furthermore, the use of polyaniline–titanium(IV)phosphate [13], polyvinyl chloride (PVC) membrane with active agent bovine serum albumin [14], and ether acridono [15] have also been reported. Another membrane used to construct Pb^2+^ ISE is G-quadruplexba used with active agent AuNPs-DNA [16]. Pb^2+^ ISE as reported in the foregoing cited literature, has a relatively narrow linear range suggesting the need for further investigation to improve the analytical performance of Pb^2+^ ISE.

Herein, the polyurethane (PU) membrane was used as a matrix, synthesized from castor oil (*Ricinus communis* L.), to construct the Pb^2+^ ISE system. The castor oil-based PU was selected due to its possession of carbonyl and amine groups resulting in a negatively charged surface [17]. Another reported study suggests that the amine group of the PU could form covalent bonds with the active agent cerium (IV) phosphate [18]. In addition, PU has a hydrophobic property as reported previously [19]. This property is beneficial for its application involving aqueous media that could maintain the membrane stability (prevent swelling and leaching of active agent).

Based on its negatively charged surface property, castor oil-based PU membrane was selected as the matrix for cationic ISE such as Pb. The selectivity of the ISE toward Pb^2+^ ions is expected to be achieved by optimizing the condition of the membrane that contains immobilized 1,10-phenanthroline. The 1,10-phenanthroline/PU membrane was immersed in Pb(NO_3_)_2_ solution to form the Pb-phenanthroline complex. The complex could form interface equilibrium on the membrane that produces interface potential that is correlated with the activity of Pb^2+^ ions in the solution. 

## 2. Materials and Methods

### 2.1. Materials

Materials used in this research included 1,10-phenanthroline, Pb(NO_3_)_2_, acetone, toluene diisocyanate (TDI), KCl, FeCl_3_, NaNO_3_, Cr(NO_3_)_3_, CuSO_4_, ZnSO_4_, Cd(NO_3_)_2_, Ni(NO_3_)_2_, Co(NO_3_)_2_, Mg(NO_3_)_2_, KNO_3_, Fe(NO_3_)_3,_ FeCl_3_, CH_3_COOLi, and Ag wire. All the aforementioned chemicals were purchased from Merck with analytical grade quality. Commercial castor oil (*Ricinus communis* L.) was procured from PT. Rudang Jaya (Medan, Indonesia) with industrial grade quality and an agar was purchased from trademark Akos. A glue with the trademark UHU has been used as an adhesive to attach the PU membrane to the surface of the electrode body. The wastewater sample was collected from the area surrounding Industrial Area II in Medan, Indonesia. 

### 2.2. Instruments

For the membrane characterization, instruments used herein included Scanning electron microscope (SEM) with a serial name Jeol Jsm 6360 LA (Tokyo, Japan), X-ray diffractometer (XRD)—Shimadzu XRD-700 Series (Kyoto, Japan), Fourier transform infrared (FT-IR) spectrometer—Shimadzu Prestige (Kyoto, Japan), and Universal Testing Machine HT8503 (Hung Ta Instrument Co., Ltd., Taichung, Taiwan). Moreover, during the analytical performance of the ISE Pb^2+^, we used potentiometer Orion model with a serial name Thermo Orion Scientific Star A2115 (Waltham, MA, USA) and atomic absorption spectroscopy (AAS)—Shimadzu AA-7000 (Kyoto, Japan). A hand-made Ag/AgCl reference electrode was also used.

### 2.3. Preparation of 1,10 Phenantrolin-Immobilized PU Membrane 

Firstly, the membrane matrix was prepared by adding 1.75 g TDI into 3.5 g castor oil in a glass beaker, and then stirring for 3 min. Thereafter, 1,10-phenanthroline was added to the mixture with weight variation of 0, 1, 3, 5, 7, and 19 mg, stirred until homogenous and heated for 15 min at 45 °C. The next process involved sonicating the mixture while adding with 4 g of acetone. The solution was then casted on a glass plate and subsequently oven dried at 40 °C for 24 h.

### 2.4. Preparation of Ag/AgCl Reference Electrode

An Ag/AgCl reference electrode was prepared through electrolysis employing two Ag wires (d = 0.57 mm), performed in KCl 0.1 M solution for 30 s. The electrolysis lasted for 30 s until the black color was formed on the wire surface indicating the formation of Ag/AgCl. 

### 2.5. Construction of Pb^2+^ Ions Selective Electrode (Pb^2+^ ISE)

**Figure 1:**
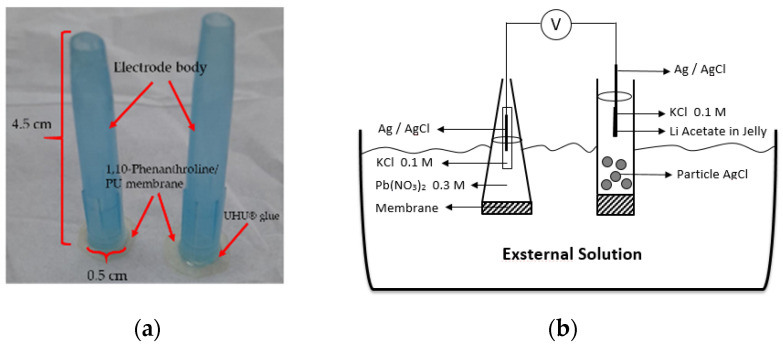
Pb^2+^ ISE-based on PU membrane (**a**). A schematic diagram of potentiometric cell (**b**).

Previously prepared membrane was cut into a round shape with a diameter of 0.57 mm and glued onto an electrode body surface. Then, the internal solution was poured which contained 0.1 M KCl and 0.3 M Pb(NO_3_)_2_. The ion selective electrode (ISE) was conditioned by soaking the electrode into a 0.1 M Pb(NO_3_)_2_ solution for 24 h. Prior to the analysis, the Pb^2+^ ISE surface was washed clean using distilled water. The measurement and optimization of the electrode were performed with 10^−10^–10^−1^ M Pb(NO_3_)_2_ standard solutions. The electrode and a schematic potentiometric cell are presented in Figure 1.

### 2.6. Optimization of Pb^2+^ISE

#### 2.6.1. Effect of 1,10-Phenanthroline Composition

The determination of optimum 1,10 phenanthroline weight was obtained based on the best sensitivity value with a broad linear range. The sensitivity of ISE was calculated from the slope at the linear region of the plots of the electrode potential (mV) and Pb(NO_3_)_2_ concentration. 

#### 2.6.2. Effect of Internal Solution Concentration

A membrane prepared with the optimum 1,10 phenanthroline composition was used to determine the effect of internal solutions consisting of KCl and Pb(NO_3_)_2_ against the ISE sensitivity and the width of linear range. The sensitivity was obtained based on the linear curve of the plot potential (mV) vs. Pb(NO_3_)_2_ concentration.

#### 2.6.3. Effect of pH 

An investigation on the effect of pH on Pb^2+^ ISE performance was conducted by measuring the potential against Pb(NO_3_)_2_ solution at pH 4—9 using phosphate buffer solution of 0.1 M. 

### 2.7. Analytical Performance Analysis of Pb^2+^ ISE

#### 2.7.1. Determination of Response Time

The response time of Pb^2+^ ISE was determined based on the minimum time required to yield constant potential (mV). It was performed on Pb(NO_3_)_2_ solutions of 10^−10^–10^−5^ M. Potential was recorded after reaching a stable value indicated by a potential change in the range of ±0.1 mV to ±0.6 mV. 

#### 2.7.2. Repeatability Test

The repeatability test of the constructed Pb^2+^ ISE was performed by determining the ISE potential value measured repeatedly using the same ISE. Thereafter, the standard deviation (SD) was calculated for the obtained potential values.

#### 2.7.3. Reproducibility Test

The reproducibility test was carried out on the potential response from ISE with optimum performance. The reproducibility value was obtained based on the SD of the sensitivity of 10 electrodes.

#### 2.7.4. Selectivity Test for Pb^2+^ ISE

The selectivity of Pb^2+^ ISE was determined by using mixed solutions. The investigation was carried out with a Pb(NO_3_)_2_ concentration of 10^−4^ M following the introduction of Ca^2+^, Ag^2+^, Fe^3+^, Cu^2+^, Mg^2+^, K^+^, Cr^3+^, Zn^2+^, and Cd ^2+^ ions with the same concentrations. 

#### 2.7.5. Determination of Lifetime

The lifetime of Pb^2+^ ISE was determined by the deviation of the first-day sensitivity value compared with the measurement obtained on the following days. The test was carried out for 25 days with 5 day intervals.

### 2.8. Characterization of 1,10-Phenanthroline-Immobilized PU Membrane

To observe the effects of the characteristics of the 1,10-phenanthroline-immobilized PU membrane on the Pb^2+^ ISE analytical performance, we performed several analyses on the membrane sample before and after use for Pb^2+^ ion measurement at optimum conditions. These analyses included FT-IR to observe the functional groups, SEM—the surface morphology, and XRD—crystallinity, and tensile strength determination.

## 3. Results and Discussion

### 3.1. Pb^2+^ ISE Optimization

#### 3.1.1. Effect of 1,10-Phenanthroline Weight 

**Figure 2:**
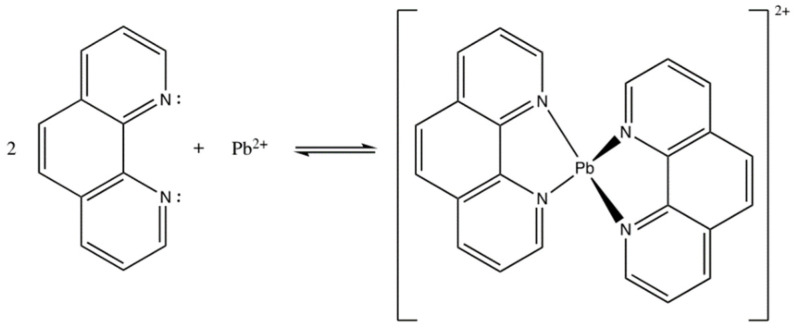
Interaction between 1,10-phenanthroline and Pb^2+^ forming phenanthroline-Pb complex.

1,10-phenanthroline acts as an ionophore or active compound that is selective in the working system of Pb^2+^ ISE. 1,10-phenanthroline has a function to bind targeted ions from the solution. However, since 1,10-phenanthroline could also be used as a ligand to bind other cations, we designed the working system of ISE using Pb containing an internal solution. The same idea was employed by Papp et al. (2018) [20] in the making of Cu^2+^ ISE using hydrophilic tripeptide as the active agent. In this present study, the ionophore 1,10-phenanthroline was immobilized into a PU membrane functioned as the sensor matrix. 1,10-phenanthroline is a ligand comprising a free electron pair on two N atoms which bind the cations. According to its binding mechanism, interaction between 1,10-phenanthroline and Pb^2+^ ions was based on Coulomb force [21], highlighting that Coulomb attraction force interaction occurs on ions with different charges. The interaction between 1,10-phenanthroline and Pb^2+^ has been illustrated and presented in Figure 2.

**Figure 3:**
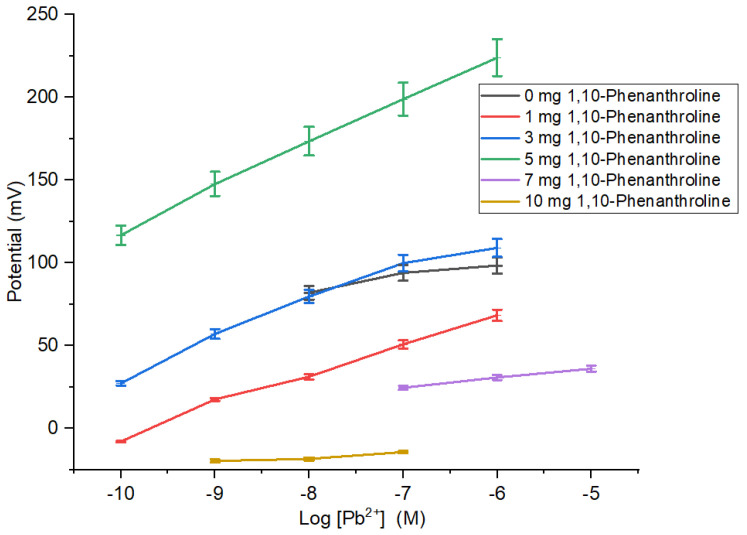
Effect of amount 1,10-phenanthroline on sensitivity and linear range of Pb^2+^ ISE.

Ionophore composition causes the characteristics of the membrane indicated by the sensitivity of ISE membrane in determining the concentration of Pb^2+^ ion (Table 1 and Figure 3). The sensitivity of Pb^2+^ ISE was close to Nernstian for two valence ions and had the broadest linear range obtained by ISE membrane added with 5 mg 1,10-phenanthroline. On the contrary, the ISE membranes with 0, 1, and 3 mg 1,10-phenanthroline had low sensitivities and narrow linear ranges. This is ascribed to the insufficient number of ionophore causing the ISE sensitivity to be unable to reach the theoretical value. The ISE sensitivity experienced a deprivation for membranes with ionophore of >5 mg. Moreover, the addition of 1,10-phenanthroline could be affected by the hydrophobicity of the PU. The hydrophobic characteristic may be attributed to the binding of active agent, hence preventing the detachment of the active agent into the analyte solution and subsequently contributes to higher ion inter-surface mobility through the membrane due to more effective ion exchange capacity [22].

#### 3.1.2. Effect of Internal Solution Concentration

**Figure 4:**
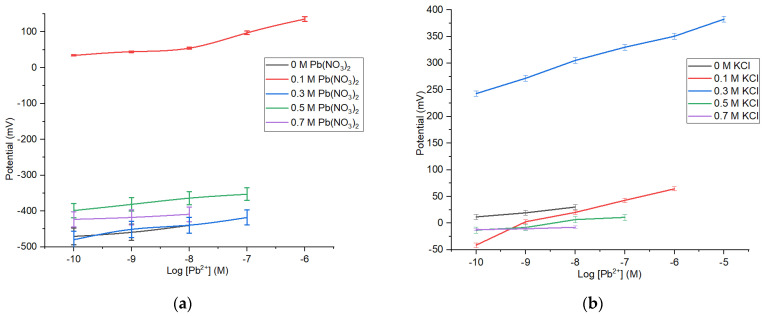
Profile of Pb^2+^ ISE sensitivities and linear ranges on the variation of internal solution Pb(NO_3_)_2_ (**a**) and KCl (**b**).

The constructed Pb^2+^ ISE is a type of ISE that uses internal solution. A solution made from Pb(NO_3_)_2_ and KCl was used as the internal solution, functioned to stabilize the electrode performance. Determination of solution potential on ISE is likely affected by the ionic strength in the internal solution [23]. The data in Table 2 and Figure 4 show that the composition of Pb(NO_3_)_2_ and KCl affects the sensitivity and linear range of the Pb^2+^ ISE.

The measurement results show that KCl concentration used as internal solution affects the sensitivity. The sensitivity of Pb^2+^ ISE is close to the Nenstian value obtained at [KCl] = 0.1 M, with a sensitivity of 25.57 mV/decade, at a Pb(NO_3_)_2_ concentration of 10^−10^–10^−6^ M. Table 2 shows that ISE without the KCl has a low sensitivity. Nonetheless, the increase in the internal solution concentration also could cause the reduction in sensitivity. 

#### 3.1.3. Effect of pH

**Figure 5:**
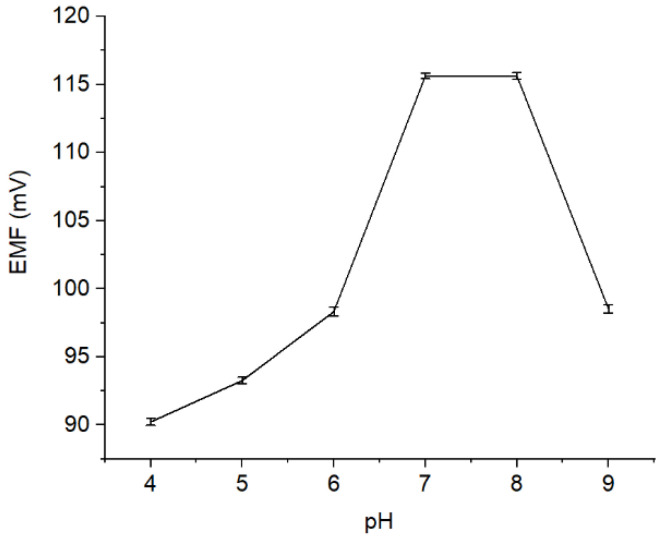
Potential curve (mV) measured on different pH levels (pH 4–9).

The effect of pH on the performance of Pb^2+^ ISE was evaluated by measuring the solution potential of Pb(NO_3_)_2_ 10^−4^ M at pH 4–9, where the results are presented (Figure 5). As observed, ISE response is stable at pH 7—8. It is owing to the fact that at pH < 7, the Pb^2+^ ionophore is deprotonated causing the disruption of its function to interact with the Pb^2+^ ion analytes [24]. Meanwhile, when the pH level was increased to pH > 8, the sample solution becomes too basic, where the Pb^2+^ is expected to precipitate [25]. Conversely, at low pH, the solution is rich in H^+^ causing the protonation. In this situation, the active site of the membrane becomes less effective, indicated by a decrease in the potential value.

#### 3.1.4. Effect of TISAB Solution

**Figure 6:**
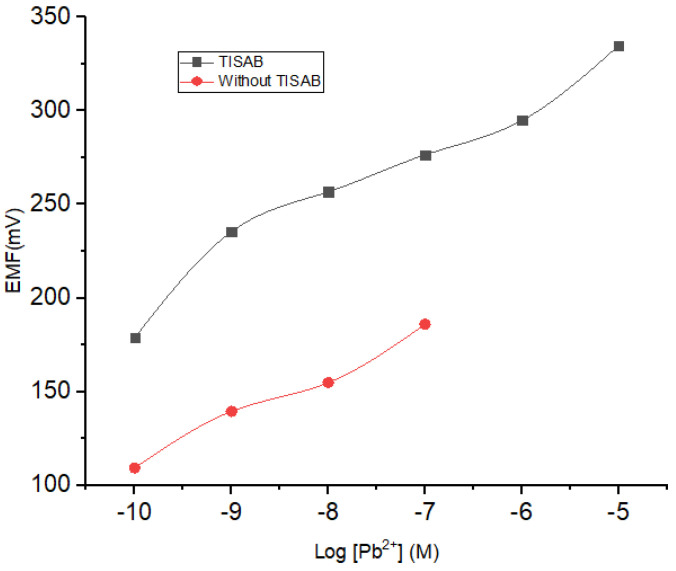
Effect of TISAB on the sensitivity and linearity range of ISE Pb^2+.^

The potential determination using ISE is dependent on the solution ionic strength. In this regard, a total ionic strength adjuster buffer (TISAB) acts to maintain the stability of the ionic strength in the solution. On the other hand, TISAB could also contribute to the results obtained from the measurement of the potential. Herein, NaNO_3_ 10^−4^ M was selected as the TISAB, mixed into each concentration of Pb^2+^ with a concentration range from 10^−10^ to 10^−1^ M. The results of the investigation on the influence of TISAB are presented in Figure 6 and Table 3. By incorporating TISAB into the analyte solution, a wider linear range was obtained which could be ascribed to its role in stabilizing the ionic strength at low concentrations.

### 3.2. Performance Characteristics of Pb^2+^ ISE

#### 3.2.1. Profiles of Sensitivity, Linear Range, and LOD of Pb^2+^ ISE

**Figure 7:**
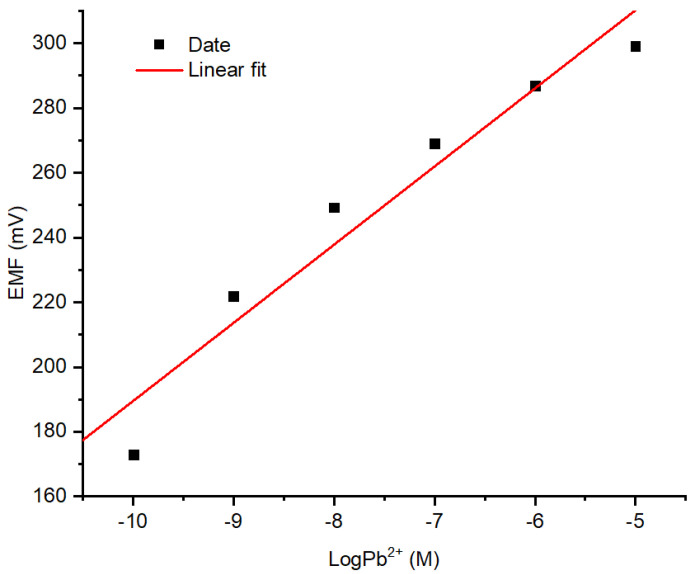
Response profile of Pb^2+^ ISE on varied concentrations of Pb(NO_3_)_2._

The responses Pb^2+^ ISE on the variation of Pb(NO_3_)_2_ concentration with the previously documented optimum conditions are presented in Figure 7. The linear range was obtained at Pb(NO_3_)_2_ concentration ranged from 10^−10^ to 10^−5^ M with an acceptable coefficient of determination value of 0.970. As can be seen from the curve, a lower concentration of Pb^2+^ ion causes lower potential (mV). This corresponds to the Nernst equation for cations as shown in the equation below:

$$Ecell=Eo+\frac{0.05916}{n}\log\left[A\right]$$

The determination of the LOD of Pb^2+^ ISE was carried out by measuring the potential of blank solution (in this regard the solution was NaNO_3_ 10^−4^ M. The LOD obtained was 10^−10^ M calculated using the equation expressed below:

$$-{\mathrm{Log}}_{\mathrm{LOD}}=\frac{Y_{LOD}-intercept}{slope}$$

#### 3.2.2. Response Time

**Figure 8:**
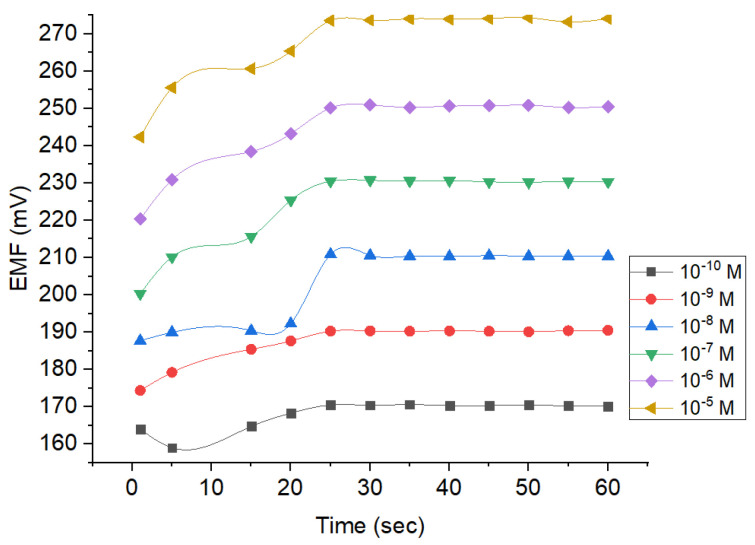
Response time profile of Pb^2+^ ISE using different analyte concentrations.

Response time is an essential parameter to determine the time required to reach the stable potential. By doing so, the data obtained would be more precise and accurate. Based on Figure 8, the response of the Pb^2+^ ion sensor reaches the stability after the 25th second and lasted until the 60th second. The measurements on the 25th second revealed the potential values of around 170, 190, 210, 230, 250, and 273 mV when the concentrations were set at 10^−10^, 10^−9^, 10^−8^, 10^−7^, 10^−6^, and 10^−5^ M, respectively. Thereafter, the values tend to be fluctuating with changes occurring no more than 0.1 mV. Thus, 60 s were obtained for the rest of the studies because the potential values were considered to be constant. At concentration of 10^−10^–10^−5^ M, the estimated potential resulted in a drift <0.9 mV/second. It suggests the ISE is sufficiently good and appropriate for the criteria by the International Union of Pure and Applied Chemistry (IUPAC), where change potential occurring within the measurement should be 1 mV/min [26].

Figure 8 also shows that all concentrations tested required the same time to reach the equilibrium on the membrane surface. It can be concluded that the ability of ion immobilization is equal across all tested concentrations. Constant potential would be reached when the Pb^2+^ ion exchange in the analyte equals to the Pb^2+^ ions in the membrane inter-surface. The concentration is correlated with the generated potential. Hence, the higher the concentration of the analyte, the higher the potential obtained. It could be attributed to the higher occurrence of ion exchange.

#### 3.2.3. Repeatability of ISE

Repeatability of Pb^2+^ ISE was tested aiming to reveal the closeness of the potential measurement value performed with repetition using the same condition. In this test, the potential of Pb^2+^ ISE was measured using standard solution Pb(NO_3_)_2_ of 10^−10^–10^−5^ M for 5 measuring series, in which the results are presented (Table 4). The test results of the repeated measurement show similar Pb^2+^ ISE sensitivity, proven by STDV of <5%. This low deviation suggests the feasibility of using the system for analytical purposes [27].

#### 3.2.4. Reproducibility of the ISE

Reproducibility is an investigation performed to observed the deviation occurring between each cathode constructed using the same condition [28]. The analyte used was Pb(NO_3_)_2_ of 10^−10^–10^−5^ M, where results from this investigation are presented in Table 5. The sensitivity of 10 Pb^2+^ ISE had a deviation standard of less than 5%. However, indeed, the potentials resulting by one electrode with another are different, probably because the homogeneity of the membrane surface deviated from one another. Nonetheless, all of the 10 tested electrodes have the same ability of responding to the Pb^2+^ presence in the solution. 

#### 3.2.5. Selectivity of ISE

One of important parameters, selectivity coefficient (Kij), was also determined in this present study, where the data are presented in Table 6. Kij depicts the ability of ISE in responding to the primary ion of interest as opposed to other foreign ions [29]. Herein, the Kij values were measured based on the mixed solution method at [Pb(NO_3_)_2_] of 10^−4^ M. The foreign cations used included K^+^, Co^2+^, Cr^2+^, Zn ^2+^, Ag ^2+^, Mg^2+^, Ca^2+^, and Fe^3+^. The test is important since the amine group from PU also has an affinity with other competing ions [30].

#### 3.2.6. Lifetime

**Figure 9:**
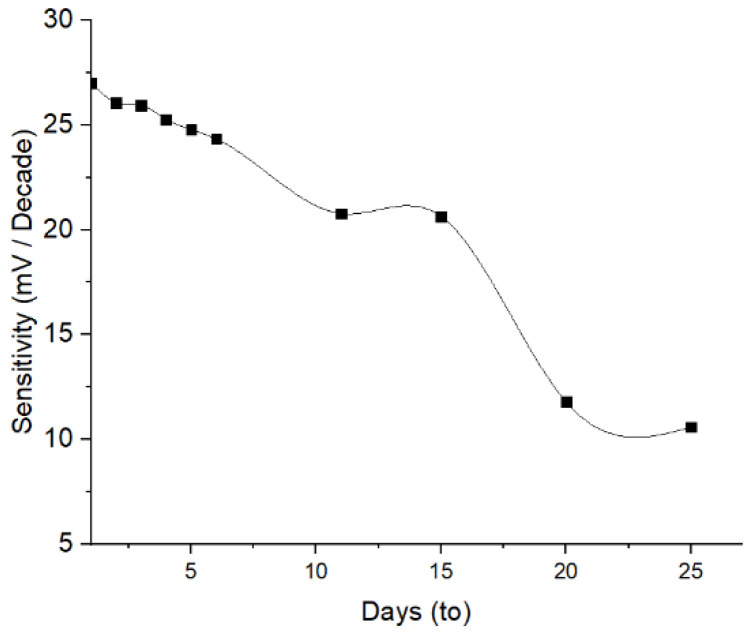
Lifetime of Pb^2+^ ISE.

The determination of the lifetime aimed to evaluate the stability of the ISE response across a variation of time. The results indicate that the Pb^2+^ ISE has a stability over 4 days of evaluation, where no significant sensitivity reduction occurred (Figure 9). The decrease in the sensitivity of 8.29% and 23.09% occurred on day-5 and -11, respectively. This reduced trend of the sensitivity probably continues after the next following days. This is ascribed to the loss of available active agent on the electrode surface owing to the membrane swelling and eventually caused leaching. This is yet unproven, since more investigation on the interaction between the PU membrane and 1,10-phenanthroline is required. Sensitivity of Pb^2+^ ISE < 25 mV/decade suggest that the system did not meet the two-valence standards [31]. A similar phenomenon was also obtained on PVC-based Pb^2+^ ISE from a previously published report [32]. 

### 3.3. Characteristics of the 1,10 Phenanthroline-Immobilized PU Membrane before and after the Pb^2+^ Measurement

#### 3.3.1. FT-IR

**Figure 10:**
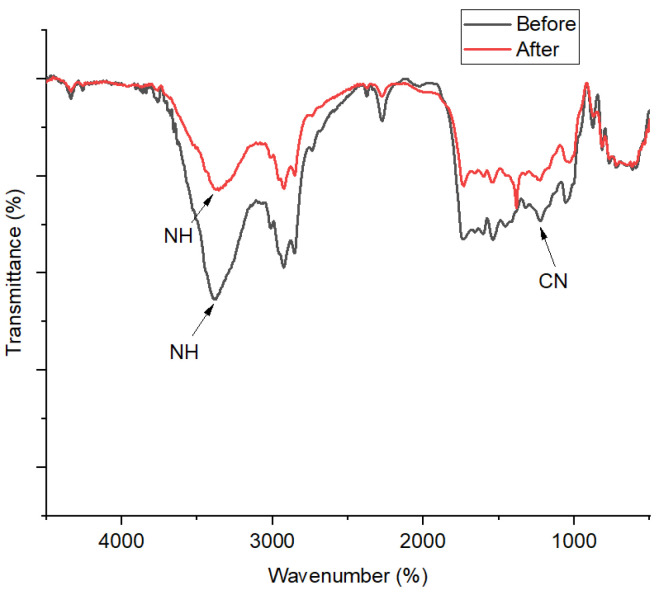
FT-IR spectra of 1,10-phenanthroline-immobilized PU membranes before and after use for Pb^2+^ analysis.

FT-IR profiles of the 1,10-phenanthroline-immobilized PU membranes before and after Pb^2+^ analysis are presented (Figure 10). A broad spectral band at 3372 cm^−1^ is assigned to the stretching vibrational band of N-H, which is formed due to the condensation of O-H from the castor oil and N=C=O from the TDI. In a previous study, the bending vibration of aromatic C-N should appear at around 1408 cm^−1^ [33]. Nonetheless, in this present study, the foregoing spectral peak was observed at 1236 cm^−1^, which indicates its disturbed vibration probably due to the interaction between 1,10-phenanthroline and the PU membrane. After Pb^2+^ ISE use, a further shift to the right of this spectral peak could be observed from 1236 to 1198 cm^−1^. A previous study suggested this kind of wavenumber shifting could be attributed to the complex interaction with metals [34]. This is corroborated by the appearance of a spectral peak at 1318 cm^−1^, assigned as ligand N-metal Pb bending vibration [35].

#### 3.3.2. SEM

**Figure 11:**
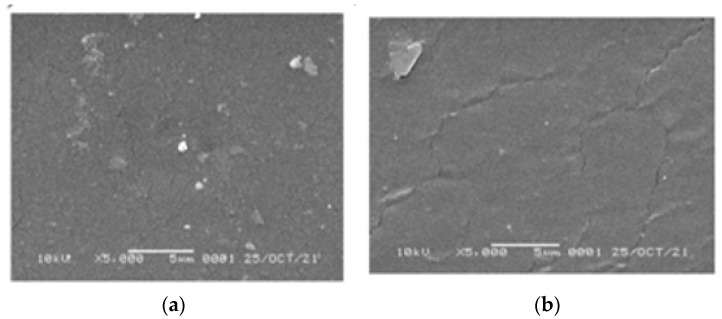
SEM images of the 1,10- phenanthroline-immobilized PU membranes before (**a**) and after use for Pb^2+^ analysis (**b**).

SEM images of the 1,10-phenanthroline-immobilized PU membranes before and after use for Pb^2+^ analysis, observed under 5000× magnification, are presented (Figure 11). The surface morphology of both membranes appeared relatively smooth and dense, suggesting the proper variation of castor oil and TDI compositions used during the preparation [36,37]. After the analysis, the crack-like structure is more pronounced on the membrane surface. Similarly, the crack-like structure was observed on the used membrane by a previously reported study [38]. Explanation of this could be attributed to the Pb^2+^ binding that disturbs the integration of membrane structure [39]. Another explanation is the possibility of membrane swelling that also could result in membrane disintegration. 

#### 3.3.3. XRD

**Figure 12:**
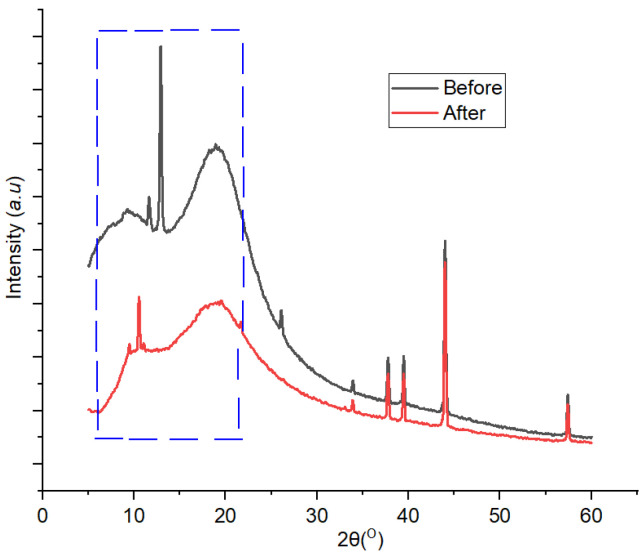
Diffractogram of 1,10-phenanthroline-immobilized PU membranes before and after use for Pb^2+^ analysis.

XRD patterns generated from the analysis of 1,10 phenanthroline-immobilized PU membranes before and after use for Pb^2+^ determination are presented in Figure 12. A clear crystallinity modification occurred after the membrane was exposed with Pb^2+^ solution and could be observed at a 2Ɵ range of 5°–15°. Attenuation of crystalline peaks in the used membrane was observable throughout the diffractogram, indicating the effect of Pb^2+^ complexation on the membrane structure which subsequently reduced its crystallinity. The crystallinity values of the membrane before and after use for the determination of Pb^2+^ were 99.87% and 88.16%, respectively. The precedence of this case has been reported [40]. Furthermore, in the used membrane, a new small crystalline peak appeared at 2Ɵ = 16°C, which could be assigned to the Pb^2+^—1,10-phenanthroline complex [41,42].

#### 3.3.4. Mechanical Properties

**Figure 13:**
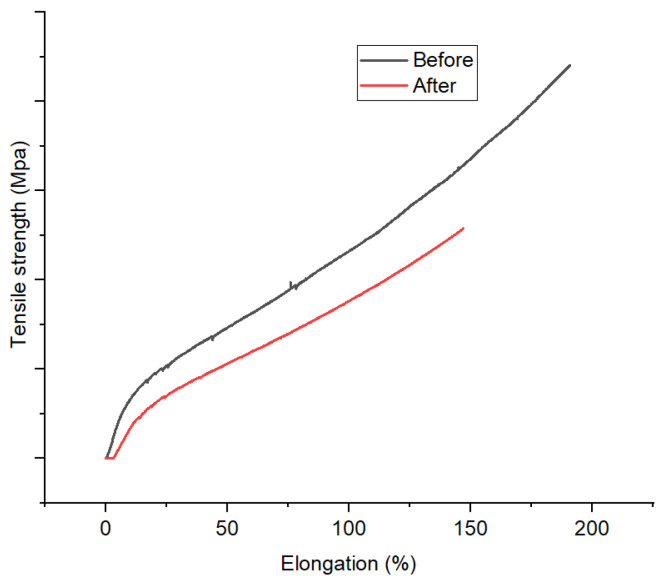
Mechanical profile 1,10-phenanthroline-immobilized PU membranes before and after used for Pb^2+^ analysis.

Mechanical profiles of 1,10-phenanthroline-immobilized PU membranes before and after use for Pb^2+^ analysis, as depicted by elongation (%) versus tensile strength (MPa), are presented (Figure 13). Mechanical properties could reveal the effect of the membrane use as Pb^2+^ ISE on its structure. Herein, a reduction in mechanical properties of the membrane was observed. A similar phenomenon has been reported by other research groups [43]. This finding corroborates the previous ones regarding the loss of membrane structure integrity after incorporating Pb^2+^ through complexation. However, the possible role of membrane swelling should not be ruled out. Overall, this analysis along with the previous ones (SEM and XRD) suggest the membrane degradation after use for Pb^2+^ analysis. This is the downside of the membrane being unable to be reused. 

### 3.4. Investigation on Real Sample

The optimized Pb^2+^ ISE was tested for its performance on two artificial wastewater samples ([Pb^2+^] = 6.8 and 7.6 mg/L) and a real sample. The real sample collected the wastewater effluent from the Medan Industrial Area, North Sumatra, Indonesia. The aim was to identify the accuracy of Pb^2+^ ISE for Pb^2+^ ion detection as compared with the standard method. The standard method used for the validation herein was atomic absorption spectroscopy (AAS). The results of this validation are presented in Table 7. By comparing the data obtained from Pb^2+^ ISE with those obtained from AAS, we obtained the results of the t-test calculation on three repetitions of measurements and show that there was no significant difference between the Pb measurements of ISE and AAS. Such value range suggests the good analytical performance of this proposed method which is close to the AAS method. 

## 4. Conclusions

The Pb^2+^ ISE constructed herein was based on PU membrane and 1,10-phenanthroline, and was successfully developed with very good selectivity and analytical performance against the working parameters of ISE. The analytical method developed had excellent accuracy and good correlation in determining the Pb^2+^ ions from the analyte solution. FT-IR analysis proved that the 1,10-phenanthroline has a primary role in Pb^2+^ ion complexation during the analysis. The downside of this membrane is the degradation of its structure integrity following its usage as Pb^2+^ ESI. Application in the real sample suggests our newly proposed method could be used to determine Pb^2+^ in wastewater.

## Figures and Tables

**Table 1:** Sensitivity and linear range profiles of Pb^2+^ ISE against the weight variation of 1,10-phenanthroline.

1,10-Phenanthroline (mg)	Sensitivity (mV/Decade)	Linear Range (M)	R^2^
0	8.19 ± 0.15	10^−10^–10^−8^	0.966 ± 0.03
1	18.45 ± 0.14	10^−10^–10^−6^	0.962 ± 0.05
3	20.58 ± 0.13	10^−10^–10^−6^	0.976 ± 0.02
5	26.46 ± 0.11	10^−10^–10^−6^	0.985 ± 0.01
7	5.71 ± 0.13	10^−7^–10^−5^	0.993 ± 0.01
10	2.68 ± 0.03	10^−9^–10^−7^	0.935 ± 0.04

**Table 2:** Sensitivity of the electrode depending on the internal standard component variation.

Standart Solution Compositions		Sensitivitas (mV/ Decade)	Linear Range (M)	R^2^
Pb(NO_3_)_2_ (M)	KCl (M)			
0.3	0	15.24 ± 0.13	10^−10^–10^−8^	0.987 ± 0.01
	0.1	25.57 ± 0.002	10^−10^–10^−6^	0.950 ± 0.05
	0.3	19.73 ± 0.16	10^−10^–10^−7^	0.978 ± 0.001
	0.5	15.40 ± 0.05	10^−10^–10^−7^	0.975 ± 0.02
	0.7	7.142 ± 0.01	10^−10^–10^−8^	0.970 ± 0.02
0	0.1	9.20 ± 0.1	10^−10^–10^−8^	0.960 ± 0.02
0.1		25.13 ± 0.03	10^−10^–10^−6^	0.990 ± 0.01
0.3		27.25 ± 0.14	10^−10^–10^−5^	0.991 ± 0.004
0.5		8.62 ± 0.06	10^−10^–10^−7^	0.973 ± 0.04
0.7		2.23 ± 0.10	10^−10^–10^−8^	0.962 ± 0.01

**Table 3:** TISAB solution comparison profile and without TISAB.

Parameters	With TISAB	Without TISAB
Sensitivity (mV/Decade)	24.04 ± 0.11	27.44 ± 0.11
Linearity range (M)	10^−10^–10^−7^	10^−10^–10^−5^
R^2^	0.950 ± 0.002	0.953 ± 0.001

**Table 4:** Repeatability of Pb^2+^ ISE with linear range Pb(NO_3_)_2_ solutions of 10^−10^–10^−5^ M.

[Pb(NO_3_)_2_]	Repeation				
	I	II	III	IV	V
Sensitivity (mV/decade)	26.819 ± 0.001	26.28 ± 0.003	27.28 ± 0.004	26.04 ± 0.01	25.38 ± 0.02
R^2^	0.994 ± 5.6 × 10^−5^	0.941 ± 3 × 10^−4^	0.94 ± 5.8 × 10^−5^	0.978 ± 1 × 10^−4^	0.991 ± 5.8 × 10^−5^

**Table 5:** Reproducibility of Pb^2+^ ISE with linear range of 10^−10^–10^−5^ M.

Parameter	Electrode Reproducibility									
	I	II	III	IV	V	VI	VII	VIII	IX	X
Sensitivity (mV/Decade)	25.29 ± 0.01	24.95 ± 0.02	25.42 ± 0.01	24.64 ± 0.03	24.65 ± 0.03	24.65 ± 0.34	25.04 ± 0.02	24.58 ± 0.08	24.26 ± 0.03	24.64 ± 0.07
R^2^	0.987 ± 5.77 × 10^−5^	0.986 ± 5.77 × 10^−5^	0.991 ± 1.5 × 10^−4^	0.992 ± 2.1 × 10^−4^	0.992 ± 5.9 × 10^−4^	0.992 ± 2 × 10^−4^	0.997 ± 5.77 × 10^−5^	0.993 ± 5.1 × 10^−4^	0.996 ± 1.1 × 10^−4^	0.996± 2.1 × 10^−4^

**Table 6:** Selectivity coefficient of the Pb^2+^ ISE against various foreign ions.

Foreign Ions	Log K_ij_
Ag^2+^	−6.14 ± 0.0002
Ca^2+^	−6.68 ± 0.02
K^+^	−7.18 ± 0.002
Mg^+^	−7.26 ± 0.005
Cu^2+^	−7.31 ± 0.005
Fe^3+^	−7.92 ± 0.007
Cr^3+^	−8.48 ± 0.002
Zn^2+^	−8.49 ± 0.02
Cd^2+^	−8.73 ± 0.004

**Table 7:** Validation of Pb^2+^ ISE.

Sample Pb(NO_3_)_2_ (mg/L)	Pb(NO_3_)_2_ (mg/L)		t-Measure	T Table
	Pb^2+^ ISE	AAS		
6.8	6.8 ± 0.04	6.6 ± 0.04	0.146	4.32
7.6	7.6 ± 0.05	7.2 ± 0.03	0.131	
Real wastewater	2.4 ± 0.09	2 ± 0.062	0.133	

Note. AAS = atomic absorption spectroscopy.

## Data Availability

All the underlying data can be obtained by request on a case-by-case basis to the corresponding author.

## References

[1] Food and Drug Administration (2015). Elemental Impurities Guidance for Industry.

[2] Tarragó O. (2015). Lead Toxicity Case Studies Environment Medicine.

[3] Murphy J. (2003). Additives for Plastics Handbook.

[4] Völz H.G., Kischkewitz J., Woditsch P., Westerhaus A., Griebler W.-D., De Liedekerke M., Buxbaum G., Printzen H., Mansmann M., Räde D. (2006). Pigments, Inorganic.

[5] Boldyrev M. (2018). Lead: Properties, history, and applications. WikiJ. Sci..

[6] Li J., Yin T., Qin W. (2015). An all-solid-state polymeric membrane Pb^2+^-selective electrode with bimodal pore C60 as solid contact. Anal. Chim. Acta.

[7] Custodio M., Peñaloza R., Cuadrado W., Ochoa S., Álvarez D., Chanamé F. (2021). Data on the detection of essential and toxic metals in soil and corn and barley grains by atomic absorption spectrophotometry and their effect on human health. Chem. Data Collect..

[8] Zaman B.T., Erulaş A.F., Chormey D.S., Bakirdere S. (2019). Combination of stearic acid coated magnetic nanoparticle based sonication assisted dispersive solid phase extraction and slotted quartz tube-flame atomic absorption spectrophotometry for the accurate and sensitive determination of lead in red pepper samples and assessment of green profile. Food Chem..

[9] Musielak M., Kocot K., Zawisza B., Talik E., Margui E., Queralt I., Walczak B., Sitko R. (2021). Ultratrace determination of metal ions using graphene oxide/carbon nanotubes loaded cellulose membranes and total-reflection X-ray fluorescence spectrometry: A green chemistry approach. Spectrochim. Acta Part B At. Spectrosc..

[10] Liu Y., Gao Y., Yan R., Huang H., Wang P. (2019). Disposable Multi-Walled Carbon Nanotubes-Based Plasticizer-Free Solid-Contact Pb^2+^-Selective Electrodes with a Sub-PPB Detection Limit. Sensors.

[11] Bühlmann P., Pretsch E., Bakker E. (1998). Carrier-Based Ion-Selective Electrodes and Bulk Optodes. 2. Ionophores for Potentiometric and Optical Sensors. Chem. Rev..

[12] Vassilev V., Tomova K., Boycheva S. (2007). Pb(II)-ion-selective electrodes based on chalcogenide glasses. J. Non-Cryst. Solids.

[13] Khan A.A., Baig U. (2012). Electrically conductive membrane of polyaniline–titanium(IV)phosphate cation exchange nanocomposite: Applicable for detection of Pb(II) using its ion-selective electrode. J. Ind. Eng. Chem..

[14] Joon N.K., He N., Wagner M., Cárdenas M., Bobacka J., Lisak G. (2017). Influence of phosphate buffer and proteins on the potentiometric response of a polymeric membrane-based solid-contact Pb(II) ion-selective electrode. Electrochim. Acta.

[15] Golcs Á., Horváth V., Huszthy P., Tóth T. (2018). Fast Potentiometric Analysis of Lead in Aqueous Medium under Competitive Conditions Using an Acridono-Crown Ether Neutral Ionophore. Sensors.

[16] Tang W., Yu J., Wang Z., Jeerapan I., Yin L., Zhang F., He P. (2019). Label-free potentiometric aptasensing platform for the detection of Pb^2+^ based on guanine quadruplex structure. Anal. Chim. Acta.

[17] Saiful M., Shaleha S., Rahmi F. (2017). Tumbuhan Jarak *Ricinus communis* L.. Sintesis Membran Poliuretan Berbasis Bahan Alam.

[18] Baig U., Khan A.A. (2015). Polyurethane-Based Cation Exchange Composite Membranes: 3 Preparation, Characterization and its Application in Development of 4 Ion-Selective Electrode for Detection of Copper(II). J. Ind. Eng. Chem..

[19] Liu D., Meyerhoff M.E., Goldberg H.D., Brown R.B. (1993). Potentiometric ion- and bioselective electrodes based on asymmetric polyurethane membranes. Anal. Chim. Acta.

[20] Papp S., Jágerszki G., Gyurcsanyi R.E. (2018). Ion-Selective Electrodes Based on Hydrophilic Ionophore-Modified Nanopores. Angew. Chem. Int. Ed..

[21] Alva S., Sundari R., Aziz A.S.A., Rashid N.A.A., Gunawan W. (2018). Development of Ammonium-Selective Electrode Based on PVC/MB28 Membrane. IOP Conf. Ser. Mater. Sci. Eng..

[22] Alreja P., Kaur N. (2016). Recent advances in 1,10-phenanthroline ligands for chemosensing of cations and anions. RSC Adv..

[23] Elgamouz A., Shehadi I., Assal A., Bihi A., Kawde A.-N. (2021). Effect of AgNPs internal solution on the sensing of mercury(II) by an ion-selective electrode based on a thiol coordination from cysteine as ionophore. J. Electroanal. Chem..

[24] Arida H.A., Al-Haddad A., Schöning M.J. (2011). New solid-state organic membrane based leadselective micro-electrode. WIT Trans. Model. Simul..

[25] Karimi H. (2017). Effect of pH and Initial pb(II) Concentration on The Lead Removal Efficiency from Industrial Wastewater Using Ca(OH)2. Int. J. Water Wastewater Treat..

[26] Huang F., Wan Z., Jin Y., Wen L. (2017). The Effects of Cyclic Isothermal Oxidation on Ir/IrOxpH Electrode and a Method to Correct the Potential Drift of Metal Oxide Electrode. J. Electrochem. Soc..

[27] Sekaran R.J., Bougie U. (2016). Research Methods for Business: A skill Building. Nuevos Sistemas de Comunicación e Información.

[28] Ganjali M.R., Norouzi P., Rezapour M. (2006). Potentiometric Ion-Selective Sensors. Encyclopedia of Sensors.

[29] Hussien E.M., Derar A.R. (2019). Selective Determination of Diclofenac and Clomiphene with a Single Planar Solid-State Potentiometric Ion Selective Electrode. J. Electrochem. Soc..

[30] Tang X., Wang P.-Y., Buchter G. (2018). Ion-Selective Electrodes for Detection of Lead (II) in Drinking Water: A Mini-Review. Environments.

[31] Mei-Rong H. (2011). Lead ion-selective electrodes based on polyphenylenediamine as unique solid ionophores. Talanta.

[32] Kamal A., Tejpal R., Bhalla V., Kumar M., Mahajan R.K. (2015). Selective and sensitive lead (II) solid-contact potentiometric sensor based on naphthalene-sulfonamide derivative. Int. J. Environ. Sci. Technol..

[33] Motawie A., Madani M., Esmail E., Dacrorry A., Othman H., Badr M., Abulyazied D. (2014). Electrophysical characteristics of polyurethane/organo-bentonite nanocomposites. Egypt. J. Pet..

[34] Liu Y., Liu Y., Xu Y., He Q., Yin R., Sun P., Dong X. (2021). Phenanthroline bridging graphitic carbon nitride framework and Fe (II) ions to promote transfer of photogenerated electrons for selective photocatalytic reduction of Nitrophenols. J. Colloid Interface Sci..

[35] Rahmi, Julinawati, Nina M., Fathana H., Iqhrammullah M. (2022). Preparation and characterization of new magnetic chitosan-glycine-PEGDE (Fe_3_O_4_/Ch-G-P) beads for aqueous Cd(II) removal. J. Water Process Eng..

[36] Khan S.U., Sultan M., Islam A., Sabir A., Hafeez S., Bibi I., Ahmed M.N., Khan S.M., Khan R.U., Iqbal M. (2021). Sodium alginate blended membrane with polyurethane: Desalination performance and antimicrobial activity evaluation. Int. J. Biol. Macromol..

[37] Norouzi A., Lay E.N., Nareh A.A., Hosseinkhani A., Chapalaghi M. (2021). Functionalized nanodiamonds in polyurethane mixed matrix membranes for carbon dioxide separation. Results Mater..

[38] Yan Q., Xin B., Chen Z., Liu Y. (2021). Preparation and characterization of flexible Polypyrrole/Zirconium carbide/Polyurethane hybrid membranes with enhanced electro-photo-thermal performance. Mater. Today Commun..

[39] Yan Q., Xin B., Chen Z., Xu J., Du X., Li Y., Liu Y., Xu L. (2021). Preparation and characterization of photothermal polyurethane/zirconium carbide fibrous membranes via electrospinning. J. Text. Inst..

[40] Carreño A., Solís-Céspedes E., Zúñiga C., Nevermann J., Rivera-Zaldívar M.M., Gacitúa M., Ramírez-Osorio A., Páez-Hernández D., Arratia-Pérez R., Fuentes J.A. (2018). Cyclic voltammetry, relativistic DFT calculations and biological test of cytotoxicity in walled-cell models of two classical rhenium (I) tricarbonyl complexes with 5-amine-1,10-phenanthroline. Chem. Phys. Lett..

[41] Chavan P.V., Pandit K.S., Desai U.V., Kulkarni M.A., Wadgaonkar P.P. (2014). Cellulose supported cuprous iodide nanoparticles (Cell-CuI NPs): A new heterogeneous and recyclable catalyst for the one pot synthesis of 1,4-disubstituted—1,2,3-triazoles in water. RSC Adv..

[42] Hosseinzadeh R., Aghili N., Mavvaji M. (2021). Synthesis and characterization of nano-cellulose immobilized phenanthroline-copper (I) complex as a recyclable and efficient catalyst for preparation of diaryl ethers, N-aryl amides and N-aryl heterocycles. Polyhedron.

[43] Song J., Huang T., Qiu H., Niu X., Li X.-M., Xie Y., He T. (2018). A critical review on membrane extraction with improved stability: Potential application for recycling metals from city mine. Desalination.

